# Advancing Understanding of Anorectal Malformations Through Microfocus Computed Tomography Imaging of Resected Material

**DOI:** 10.1016/j.gastha.2025.100633

**Published:** 2025-02-03

**Authors:** Daniël Docter, Bernadette S. de Bakker, Jaco Hagoort, Joris J.T.H. Roelofs, Sjoerd de Beer, Ernst van Heurn, Joep Derikx, Marc A. Benninga, Justin de Jong, Ramon R. Gorter

**Affiliations:** 1 Department of Pediatric Surgery, Emma Children’s Hospital, Amsterdam UMC, Location AMC, Amsterdam, The Netherlands; 2 Department of Obstetrics and Gynecology, Amsterdam UMC, Location AMC, Amsterdam, The Netherlands; 3 Amsterdam Reproduction and Development Research Institute, Amsterdam, The Netherlands; 4 Amsterdam Gastroenterology Endocrinology Metabolism, Amsterdam, The Netherlands; 5 Department of Pediatric Surgery, Erasmus MC – Sophia Children’s Hospital, University Medical Center Rotterdam, Rotterdam, The Netherlands; 6 Department of Medical Biology, Amsterdam UMC Location AMC, Amsterdam, The Netherlands; 7 Department of Pathology, Amsterdam UMC Location AMC, University of Amsterdam, Amsterdam, The Netherlands; 8 Amsterdam Cardiovascular Sciences Research Institute, Amsterdam, The Netherlands; 9 Department of Pediatrics, Amsterdam UMC Location AMC, Amsterdam, The Netherlands

**Keywords:** Microfocus Computed Tomography, Histology, Simple Anorectal Malformations, Low Type Anorectal Malformations, Pediatric Surgery

## Abstract

**Background and Aims:**

Patients born with anorectal malformations (ARMs) might experience constipation and fecal incontinence. During ARM surgery (anterior and posterior sagittal anorectoplasty procedure), the distal part of the bowel (fistula) is usually resected. Microfocus computed tomography (micro-CT) imaging, capable of imaging samples in ultra-high 3-dimensional resolution, can be used to learn from this resected material. Through this technique, we aim to investigate whether or not structures, such as the internal anal sphincter (IAS), are present within this fistula.

**Methods:**

Pediatric patients undergoing surgical reconstruction for ARM were eligible for inclusion. Resected fistulas were fixed using 4% paraformaldehyde and stained with 3.75% B-Lugol for 48 hours to improve soft tissue contrast. Scans were performed on a Phoenix Nanotom micro-CT with a voxel size between 4–6 μm. Samples were destained for subsequent histopathological examination. Outcomes were presence of structures like the IAS, epithelial transition zone and ganglia. ARM fistulas were compared with a fetal anal canal sample derived from the Dutch Fetal Biobank.

**Results:**

Eleven ARM fistulas were analyzed. All samples showed evidence of normal development of the rectal wall. Columnar epithelium and stratified squamous epithelium were observed. Muscle fibers were present, arranged in circular pattern that expanded toward the distal end, becoming the intrinsic sphincter (IAS). Ganglia were present with normal appearance.

**Conclusion:**

We present micro-CT imaging to research resected material to provide new insights in microscale anatomy. The fistula, currently resected during surgical reconstruction for ARM, contains vital structures like the IAS, normal epithelial transition zone and normal ganglion cells. Although clinical functionality should be studied in the future, our results indicate that the fistula has a normal anal canal morphology and should be spared during ARM reconstruction if possible.

## Introduction

Anorectal malformations (ARMs) constitute a group of rare congenital anomalies involving the anorectum, encompassing a spectrum of malformations. In affected children, proper development of the anorectum is impaired, resulting in misplacement of the anal aperture from its normal anatomical location; centrally within the external sphincter. The opening instead is translocated (eg to the vestibule or peritoneum) or adheres to a neighboring structure (eg prostate, bladder, or vagina) through a fistula. Based on the location of this fistula, the observed anomaly can be classified according to Krickenbeck.^2^ The estimated incidence of ARMs ranges from 2 to 6 per 10,000 births.^3^ In children with ARM, normal passage of stool may not be possible and patients may experience long lasting constipation and fecal incontinence even with proper (surgical) treatment.^4^ Management of this anomaly has been challenging for both patients and doctors. With the arrival of the anterior and posterior sagittal anorectoplasty (ASARP and PSARP, respectively) in 1981, a major stride was taken in managing this condition, but the majority of patients still suffer lifelong sequelae such as defecation problems.^5^ Despite the progress in medical science, ARMs remain inadequately understood due to their rarity and diverse presentation, including variations in the location and presence of fistulas.^4^ This merits further investigation to improve patient outcomes.

Current best practices for managing ARMs depend on the specific type of malformation. To restore normal anatomy, the majority of patients undergo surgical correction through ASARP or PSARP.^6^ In these procedures, the fistula is dissected and the anorectum repositioned within the center of the external sphincter to restore its anatomical integrity.^4^^,^^5^ In many patients, the distal part of the fistula is resected to prevent anorectal prolapse and due to the idea that the fistula is an abnormal structure which would not suffice as anal canal.^7^^,^^8^ Although this procedure restores normal anatomy, functional and long-term outcomes vary considerably between patients and are hard to predict. Continence rates and frequently encountered defecation problems vary considerably based on the severity of the malformation and presence of associated anomalies (eg VACTERL-associated anomalies).^4^ In previous studies, there is discrepancy in the frequency of incontinence in low type ARMs (rectoperineal or vestibular fistulas) but figures of up to 40% are found in some studies,^9^ which becomes even higher in high type or more complex ARMs (such as recto-vesicle, urethral, prostatic or vaginal fistulas). These complains are often attributed to overflow incontinence from constipation.^9^^,^^10^ In practice, fecal incontinence due to impaired sphincter function is less frequently observed in low type ARM and more in complex or high type ARM with sacral or spinal anomalies.

If we wish to improve clinical outcomes in ARM patients, we must take into account the potential iatrogenic damage occurring during surgery. Extensive dissection with possible nerve damage and resection of the distal part of the fistula might negatively impact patient outcomes. Previous studies applying histopathological examination of the resected fistula reported contrasting results. Studies either emphasized disorganized muscle fibers or lack of ganglion cells ^7^^,^^8^ or found the fistula to represent a normal anal canal.^11^ It should be noted that in normal anatomy, the area around the internal sphincter is considered a hypoganglionic zone (1 cell per mm^2^) and ganglions could therefore be missed on histological sections,^12^ which do not represent the entire 3-dimensional make-up of the bowel and sphincter complex.

To take new strides into improved understanding of the microanatomy, microfocus computed tomography (micro-CT) emerges as a promising imaging technique for resected samples. Originally developed in collaboration with geological and industrial sciences, micro-CT allows for the nondestructive 3-dimensional visualization of samples with an ultra-high resolution of up to 1 μm volume pixel (voxel) size. In comparison, clinical CT with a focus on minimal radiation exposure and fast scans, typically achieves voxel sizes of 500–1000 μm. Micro-CT can provide detailed images of ex-vivo samples with resolution comparable to histological sections, in 3 dimensions. Other than techniques such as scanning electron microscopy, these scans are nondestructive and can be performed in a timely manner (hours or even in minutes) in both small samples as well as samples of up to 30 cm.^13^ This technology is gaining momentum in (bio) medical research, especially in the study of fetal development, where it has proven to be a valuable tool for anatomical evaluation.^14^^,^^15^

In this study, we aim to bridge the knowledge gap surrounding the fistula in ARM cases by imaging it in 3-dimensional microscale resolution using contrast-enhanced micro-CT, to understand its morphology and bring new arguments for possible preservation of this fistula. We highlight micro-CT imaging as a new exciting technique for studying surgical anatomy and colon morphology complementary to classical histology.

## Methods

### Sample Acquisition

Between September 1, 2022, and September 1, 2023, all patients referred to the Emma Childrens Hospital of Amsterdam UMC that underwent ASARP or PSARP to treat their ARM were eligible for inclusion. The Emma Childrens Hospital is accredited by the national authority as center of expertise for ARM and is a member of the European Reference Network (eUROGEN). ASARP or PSARP was performed by at least 2 pediatric surgeons specialized in reconstructive surgery for anorectal malformations. Resection of the fistula (although kept to a minimum) was performed as standard procedure and stored in 4% paraformaldehyde (PFA) directly after surgery. The samples were fixed for 48 hours.^1^ Initially, 4 longitudinally cut hemicircumferential samples were acquired and divided between micro-CT scanning and pathological follow-up. This procedure was altered to include full circumferential samples for micro-CT analyses followed by histological analysis of the same sample, as this provided more information and was technically feasible. For healthy control, a fetal sample, gestational age 22+3 weeks, with 22q11 deletion was obtained through the Dutch Fetal Biobank.^1^ It is not expected that the 22q11 deletion affects anal development.^16^^,^^17^ The complete fetus was fixed in 4% PFA for 96 hours and stored in 0.2% PFA at 6 °C until requested and approved for research. The anal canal was carefully dissected by an experienced pediatric surgeon to contain the area of interest and consequently underwent the same methodological testing as the ARM samples.

### Sample Preparation and Micro-CT Scanning

To improve soft tissue contrast, samples were stained using 3.75% B-Lugol for 48 hours.^1^^,^^18^ Samples were then rinsed in 1× phosphate buffered saline (PBS) to remove excess B-lugol.^14^ To prevent movement during scanning, samples were mounted in 1.5% agarose gel.^1^ Scanning was performed using a GE Phoenix Nanotom M Tomographer (General Electric, Wunstorf, Germany) available through the ContrasT team in Woluwe, Belgium. Due to varying sizes of the samples (hemicircumferential or full circumferential), the scan protocol was individually optimized to gain the highest image quality.^13^ Scan parameters can be found in Appendix 1.

### Histology

After scan acquisition, all samples were stored in PBS with 0.2% PFA for destaining. Four weeks submersion of the sample in PBS leads to adequate destaining of the sample while the PFA prevents infection and decompensation.^14^ Destained samples were embedded in paraffin after which 4-μm thick sections were cut. Sections were stained using hematoxylin and eosin and evaluated by an experienced pediatric pathologist. Samples were considered normal if the following criteria were met.

#### Epithelia

Two types present, intestinal columnar epithelium containing goblet cells, transitioning to stratified squamous epithelium.

#### Innervation

Nerve bundles of varying size, and presence of ganglion cells without dysmorphic features.

#### Vasculature

Arteries and veins of varying size are present without abnormal features other than typical surgically induced trauma (Hematomas).

#### Muscles

Muscle fibers arranged in circular and/or longitudinal orientation in the respective bowel wall layers without signs of hypertrophy or atrophy.

#### Submucosa

Situated between the muscularis mucosae and circular muscle layer, containing innervation and vascularization without signs of sclerosis.

### Micro-CT Analyses

Histology and scans were compared and evaluated by a panel of experts, consisting of a micro-CT analyst, a pathologist, pediatric surgeons, and an embryologist. The fetal sample was considered as healthy control and morphological aspects of its tissues were analyzed to serve as benchmark. ARM samples were individually analyzed and compared to the benchmark. Outcomes were the same as for histology and followed the same criteria.

To determine if an intrinsic sphincter was present, the circular muscle layer within the sample was analyzed to determine if it expanded toward to distal end. A segmentation analysis was performed if the resected sample included the circular muscle layer within the wall of the fistula that was clear of the resection border in a full axial slide. The muscle fibers in the sample were then semiautomatically segmented using the watershed tool in Avizo Amira 2023.1 software. The segmented data were used to create a thickness map of the structure using the same software. The median thickness of the muscle layer was compared between different height levels of the fistulas and the fetal sample.

## Results

### Samples

A total of 11 ASARP or PSARP procedures were performed between September 1, 2022, and September 1, 2023, for ARM at Amsterdam UMC. All patients were included in this study. Four hemicircumferential samples were included and 7 full circumferential. The other half of the 4 hemicircumferential samples had to be utilized in clinical histopathological examination. Demographic data of patients can be found in Table 1.

**Table 1:** Demographic Data of Patients Undergoing Surgery for ARM and Fetal Sample

ARM sample	Type of ARM	Type of surgery	Associated anomalies	Age at surgery (mo)	Sex (M/F)
ARM 1	Rectoperineal	ASARP	Caudal regression syndrome persisting notochorda, Tethered cord syndrome Ventricular septum defect, Neurogenic bladder	7	F
ARM 2	Rectovestibular	ASARP	Ventricular Septum defect	3	F
ARM 3	Rectourethral	PSARP	Hypospadia Left Crossed fused ectopia 2 left kidneys Inguinal hernia Non scrotal testis Prematurity	3	M
ARM 4	Rectovestibular	ASARP	None	3	F
ARM 5	Rectovestibular	ASARP	None	4	F
ARM 6	Rectoperineal	ASARP	None	4	F
ARM 7	Rectoperineal	ASARP	None	4	M
ARM 8	Rectovestibular	ASARP	None	3	F
ARM 9	Rectovestibular	ASARP	None	3	F
ARM 10	Rectovestibular	ASARP	Prematurity	4	F
ARM 11	Rectovestibular	ASARP	Sacral agenesis syndrome, Cervical spine stenosis, Caudal regression syndrome	4	F
Fetal Sample	DFB reference	Dissection	Condition	Gestational age	Sex
FS1	TOP124	Anal Canal and musculature	22q11 deletion	22+3 wk	M

DFB, Dutch Fetal Biobank.

### Micro-CT

**Figure 1:**
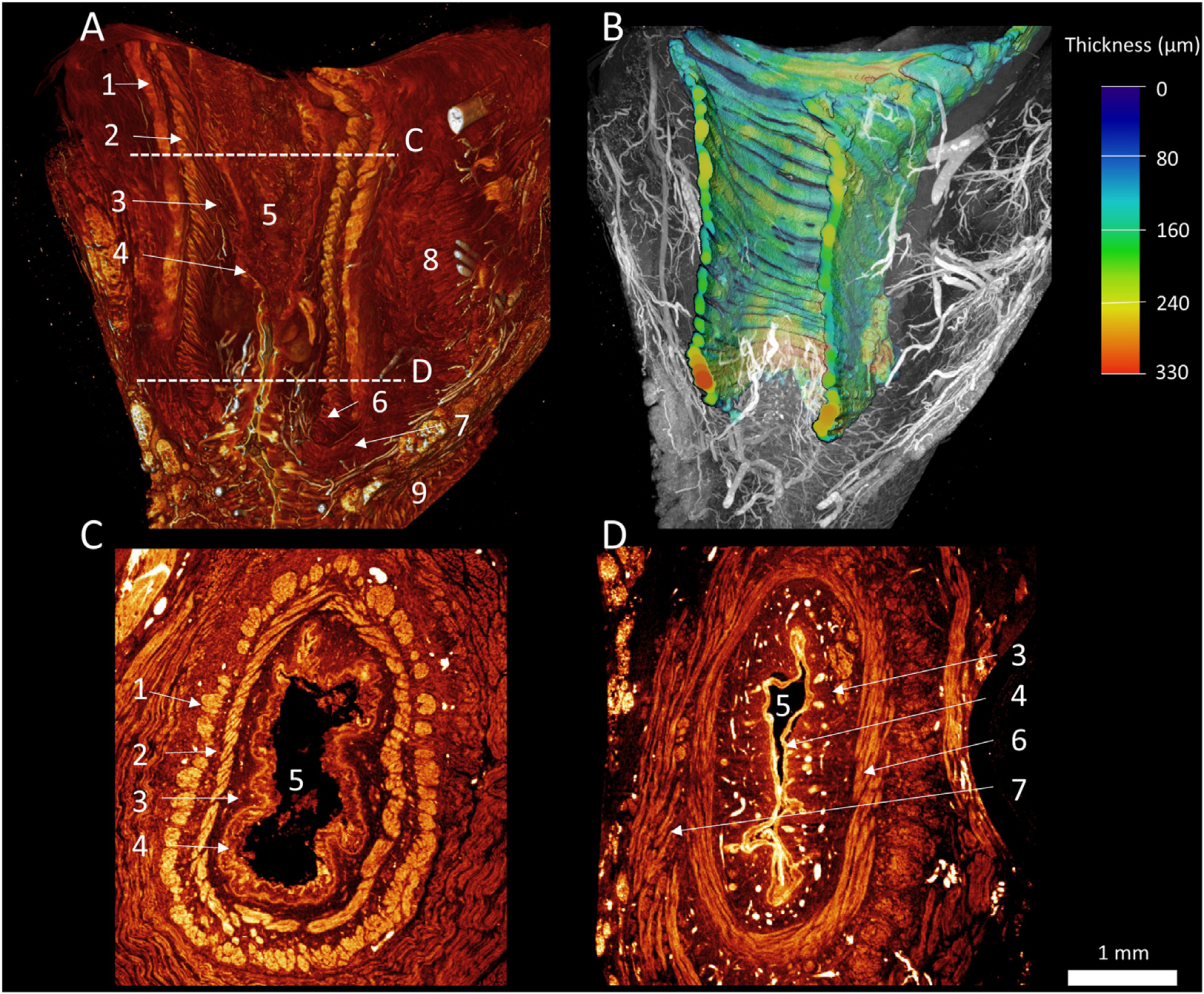
Micro-CT scan of fetal sample. (A) Rendering of the fetal scan, sagittal slice through 3-dimensional rendering to visualize the anal canal. Lines corresponding with axial slices C and D. (B) Rendering of segmented circular muscle layer, rendering corresponding with A. Circular layer can be seen to increase in size distally, becoming the internal anal sphincter. (C) Axial slice of proximal part of anal canal. A thin circular muscle, surrounded by longitudinal muscle layer and Immature villi can be observed. (D) Distal axial slice, circular muscle layer thickens and becomes internal anal sphincter. Longitudinal muscle fibers fade out. 1. Longitudinal muscle layer. 2. Circular muscle layer. 3. Submucosa 4. Epithelia. 5. Lumen. 6. Internal anal sphincter. 7. External anal sphincter. 8. Levator ani. 9. Skin.

**Figure 2:**
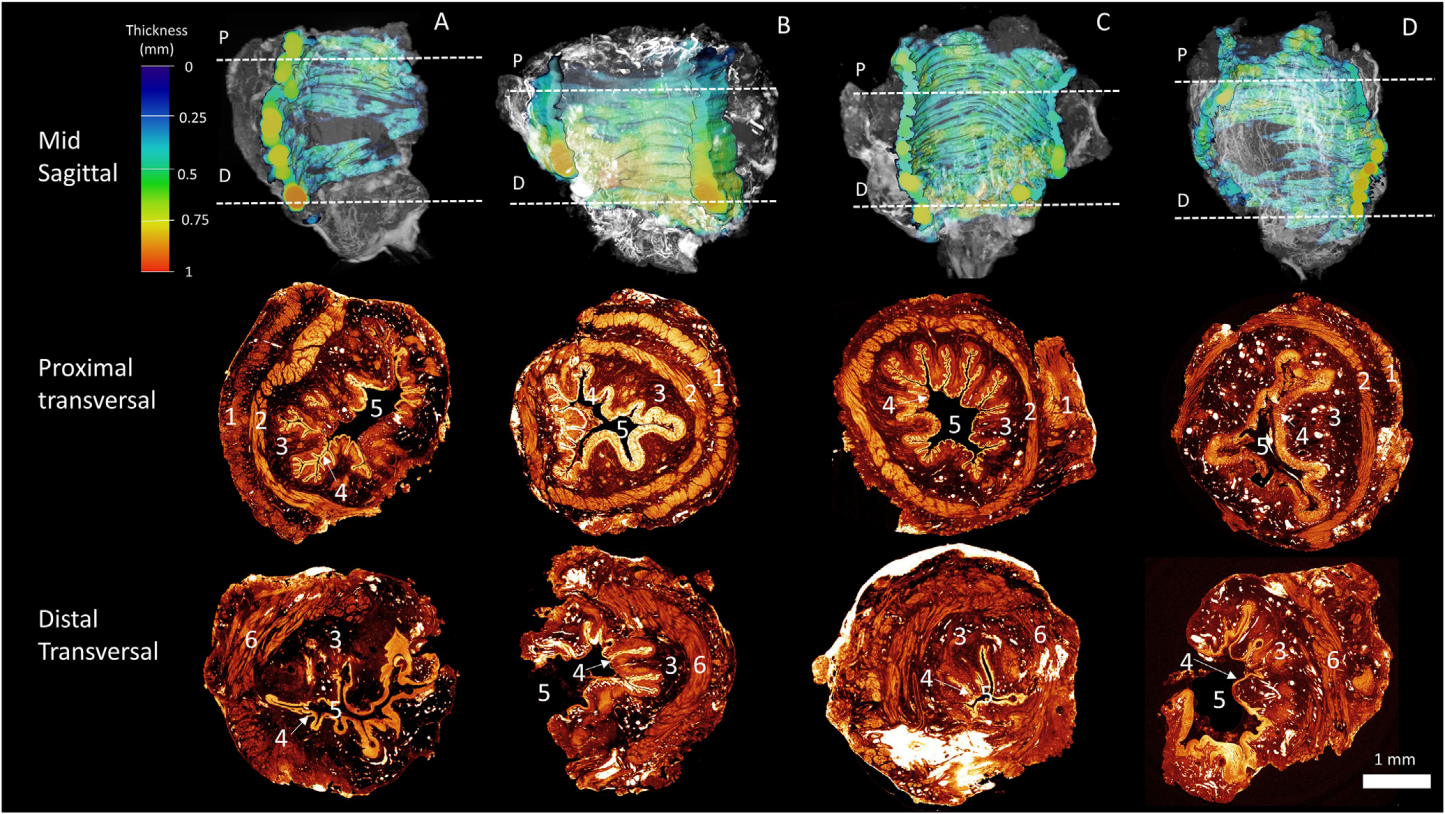
Analyses of ARM samples. Top: a sagittal cut of a 3-dimensional rendering of each sample is shown to visualize the difference in circular muscle thickness on different heights in the sample (thickness map). Middle: Proximal slices, place was determined 3 mm from the proximal end of the sample. Bottom: Distal axial slices, place was determined by the disappearance of the longitudinal layer. (A) ARM 5, resection margins are extremely close to the circular layer and much of this layer is not present in the resected sample. One side has a larger margin that also includes the longitudinal layer. (B) ARM 6, distally half of the circular layer is not included in the sample. Proximal the circular layer is fully included. (C) ARM 8, almost all of the circular layer is included in the sample apart from a small section in the distal part. (D) ARM 9, the circular layer shows holes where the dissection plane went through the circular layer. 1. Longitudinal muscle layer. 2. Circular muscle layer. 3. Submucosa. 4. Epithelia. 5. Lumen. 6. Internal anal sphincter. D, distal; P, proximal.

All samples were successfully imaged through micro-CT at between 4 and 6 μm isotropic voxel size resolution (Figure 1, Video 1). All samples had surgical damage, with the resection plane varying from medial of the circular, to just outside of the longitudinal muscle layer. All samples included either parts, or most of the circular muscle layer. In 8 samples, longitudinal fibers could be observed as well. In the 3 samples without longitudinal fibers, the resection plane was within either the circular muscle layer or the submucosa. None of the samples had resection planes lateral of the longitudinal muscle in a full single axial slide, as a cut was always present within this layer. Sutures could be identified on the scan and represented the distal end of the fistula. Toward the distal end, a change in epithelium could be observed (Figure 2). On the proximal end of samples, villi could be identified with crypts. Toward the distal end of the fistula, the epithelium transitioned into stratified squamous epithelium in all ARM samples and the fetal sample. Muscle fibers were present in every sample, arranged in a circular pattern around the epithelium of the lumen. Vessels and nerves could be visualized but were difficult to distinguish by micro-CT when reaching a diameter less than 30 μm. The fetal sample included a much wider margin than the ARM samples. This sample included the levator ani muscle and the external anal sphincter. The absolute size of the structures was smaller than in the ARM samples, in line with the age difference. Structures like the crypts were still immature (Figure 1).

### Histology

**Figure 3:**
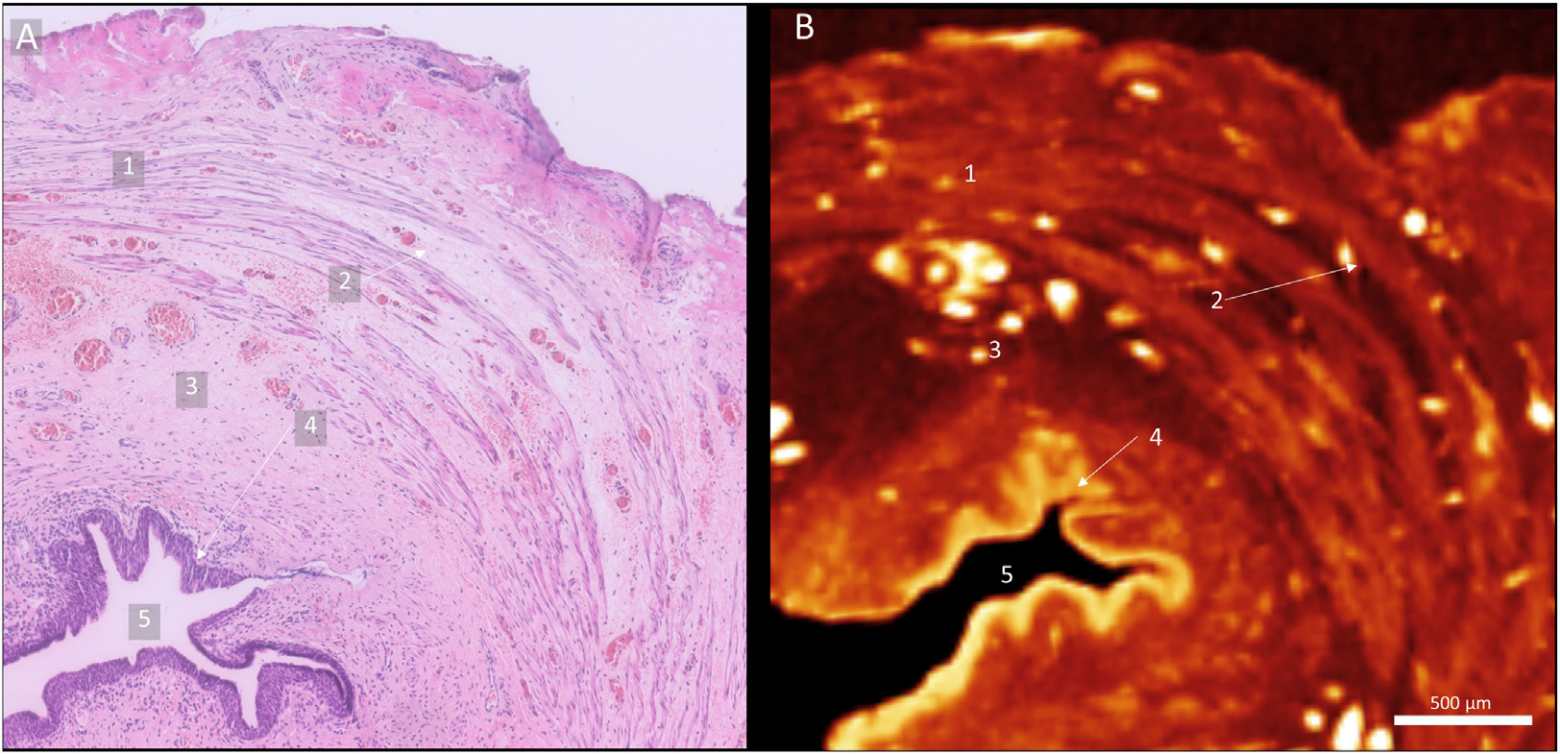
Abnormal findings. Slices of ARM 7 showing connective tissue within the circular layer. (A) Histological section. (B) Micro-CT slice corresponding to the histological slide. Both show connective tissue within the muscle fibers of this samples. 1. Circular Muscle Layer. 2. Connective tissue inside circular muscle layer. 3. Submucosa. 4. Epithelia. 5. Lumen.

Histological analyses concurred with the micro-CT findings. In all ARM samples, 2 types of epithelia were identified. Innervation, including nerve bundles of varying diameter and ganglion cells, as well as arteries and veins were present in all samples and were considered normal. All samples contained muscle fibers arranged in a circular layer. In 8 samples, partial segments of the longitudinal muscle layer were present as well. Two samples contained connective tissue within the circular layer, which was indicated as an abnormal finding (Figure 3). An overview of histological findings can be found in Table 2.

**Table 2:** Histology Results of ARM Samples

	Epithelia	Innervation	Musculature	Vascularization	Submucosa	Notes
ARM 1	Normal	Normal	Normal	Normal	Normal	Hemi circumferential
ARM 2	Normal	Normal	Normal	Normal	Normal	Hemi circumferential
ARM 3	Normal	Normal	Normal	Normal	Normal	Hemi circumferential
ARM 4	Normal	Normal	Normal	Normal	Normal	Hemi circumferential
ARM 5	Normal	Normal	Normal	Normal	Normal	Full circumferential
ARM 6	Normal	Normal	Normal	Normal	Normal	Full circumferential
ARM 7	Normal	Normal	Abnormal	Normal	Normal	Full circumferential, Connective tissue present between muscle fibers
ARM 8	Normal	Normal	Normal	Normal	Normal	Full circumferential
ARM 9	Normal	Normal	Normal	Normal	Normal	Full circumferential
ARM 10	Normal	Normal	Abnormal	Normal	Normal	Full circumferential, Connective tissue present between muscle fibers
ARM 11	Normal	Normal	Normal	Normal	Normal	Full circumferential

### Segmentation Analyses

Four samples had circular muscle fibers within the dissection plane on one or more full axial slides that could be segmented and analyzed. All of these samples showed expanding of the circular muscle layer toward the distal end from 0.1 mm proximal to 0.9 mm distal (Figure 1). Moreover, the anal canal in these samples seems to be curved toward one end. The fetal sample showed expanding of the circular layer from 0.1 mm proximal to 0.3 mm distal (Figure 2).

### Micro-CT and Histology Comparison

**Figure 4:**
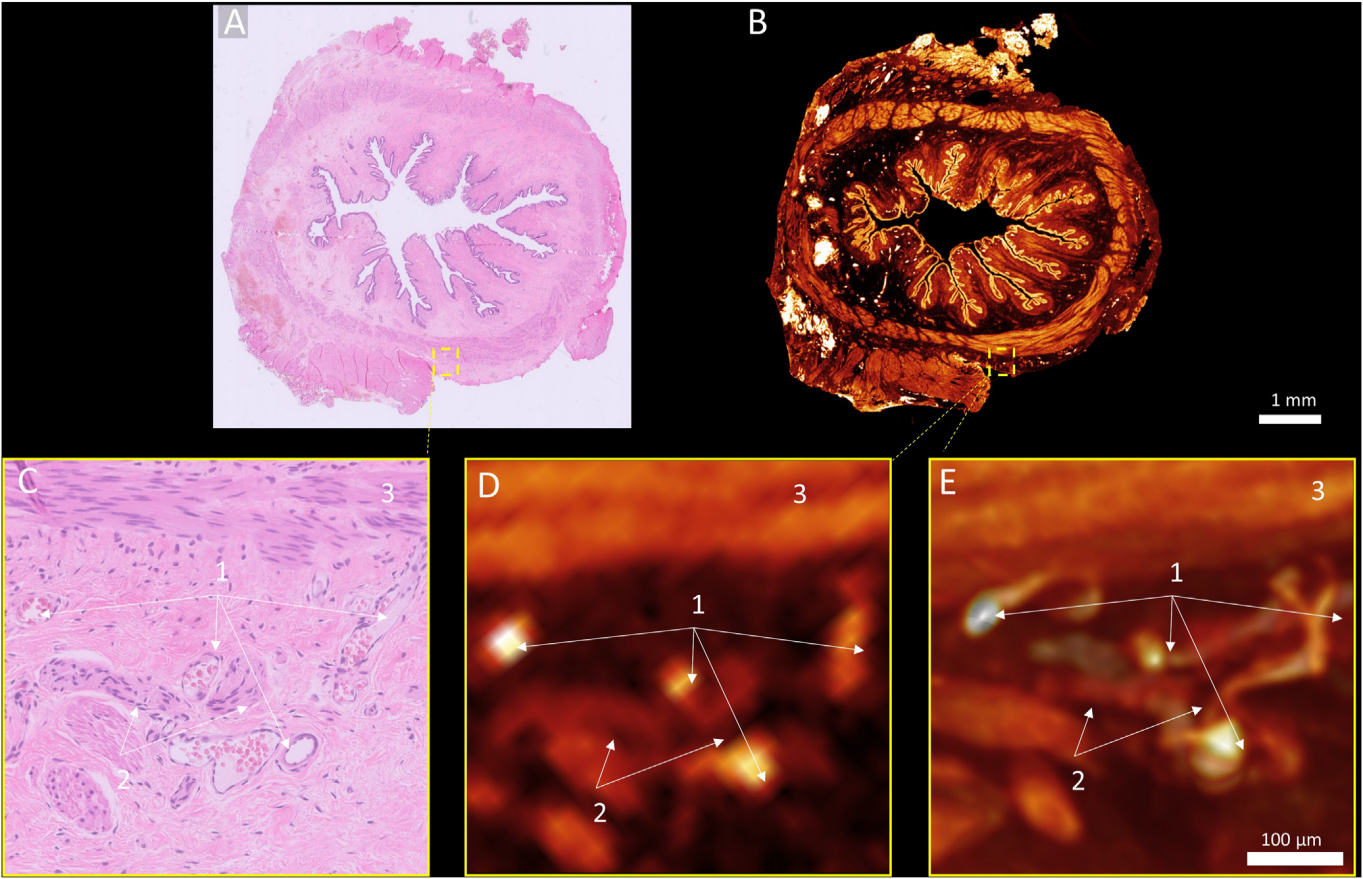
Side to side comparison of micro-CT and histology. (A) Histological section of ARM 8, Mark indication location of zoomed in section C. (B) Micro-CT axial slice of ARM 8. Axial slice is the same as the histological slide A. Mark indicating location of zoomed in section D and E. (C) Zoomed-in region of histological slide. (D) The same single slice as C of area of interest in the micro-CT scan, seeing the same vessels and nerves as in C. (E) Three-dimensional rendering of the same area of interest as C and D, showing the path of the vessels and nerves. 1. Vessels. 2. Nerves. 3. Circular muscle layer.

All histological sections were registered with the micro-CT scans to verify identified structures (Figure 3). All structures previously identified on micro-CT were in concordance with histology (Figure 4). The histological references made it possible to identify the smaller nerves, which were less dense than the blood vessels. This allowed for identification of these smaller structures in other slides as well, without the requirement of corresponding histological sections.

## Discussion

The aim of this study was to shed new light on the morphology of the fistula in ARM and determine the presence of crucial elements, such as the internal anal sphincter (IAS) through micro-CT imaging. The pathogenesis of ARM is still not fully understood and has been suspected to be caused by failure of the urorectal septum to divide the embryologic cloaca. More recent studies attribute ARM to cloacal membrane dysfunction.^4^^,^^19^ What we refer to as the fistula might in fact not be a fistula. If the problem evolves from the cloaca instead of the rectum, the rectum should be normal in morphology, albeit in a different location. As such, it may contain the relevant structures that can normally be found in the anal canal, such as the IAS, which we demonstrate here. The fistula in ARM might therefore rather be referred to as an ectopic anal orifice and depending on functionality, be treated as such.

### Novel Results from Current Study

All samples had evidence of anal canal elements on micro-CT and histology. Although the circular layer was damaged in our samples during surgery, the morphology of the circular muscle layer on micro-CT was similar in ARM cases and the fetal sample, enlarging in the most distal part to form the IAS. With histology, nerves and ganglia could be distinguished and appeared as normal innervation of the musculature. This means that the ARM fistula appears as the anal canal in terms of its microscale structure. The fistula might therefore function as such, if saved during surgery. It should be noted that although we could only quantify this in 4 samples due to surgical damage to the sample, micro-CT scans of all samples showed indication of thickening of the circular muscle layer toward the distal end. This end of the fistula is usually attached to another organ or structure, which has to be preserved, due to which the dissection plane is close on the lumen of the fistula. This is especially challenging in rectovestibular fistulas where the anterior margin would consist of mucosa. The dissections planes and the layers they represent should be taken into account if the fistula is spared during surgery.

Abnormal findings of the samples included connective tissue present between the muscle fibers of the circular muscle layer in 2 of 11 of the samples (Figure 3). The implication of this is unknown and future studies could determine the prevalence of this finding in ARM and healthy controls and determine if it impairs functionality.

### Comparison with Previous Studies

In the classic PSARP as developed by Pena in 1981, the distal part of the fistula is excised.^5^ The notion exists that this fistula is pathological or at least nonfunctional, which has been supported by findings of abnormal elements using histology.^7^^,^^8^ Similar histological studies have had opposite results, indicating that the fistula in ARM appears similar as a normal anal canal.^11^ Moreover, studies using manometric testing found normal resting pressures in the fistulas, fitting with a developed IAS 20, 21, 22 After surgery, preserving the fistula, the resting pressure was maintained and the patients were free of incontinence.^21^

In terms of innervation of this section, anatomy teaching suggests that anal sensibility is facilitated by the inferior rectal nerve, reaching the anal canal through the pudendal canal.^23^ This would suggest that anal sensibility is lost when dissecting the anal canal and moving it to the external sphincter. However, this is only the case in 30% of cases.^22^ As innervation remains intact in most patients, the anatomy of the nerves must at least partially be different than current teachings and possibly runs within the submucosa of the anal canal.^22^ Thus, the anal canal remains innervated even after relocation.^22^ Although we did not quantify this in this research, most innervation and vascularization appears to be in the submucosa or between the muscle layers on micro-CT and we did not see many crossing fibers coming from the serosa or more lateral (Figure 1). Micro-CT imaging could prove helpful in deciphering the anatomy of these structures in future research.

**Figure 5:**
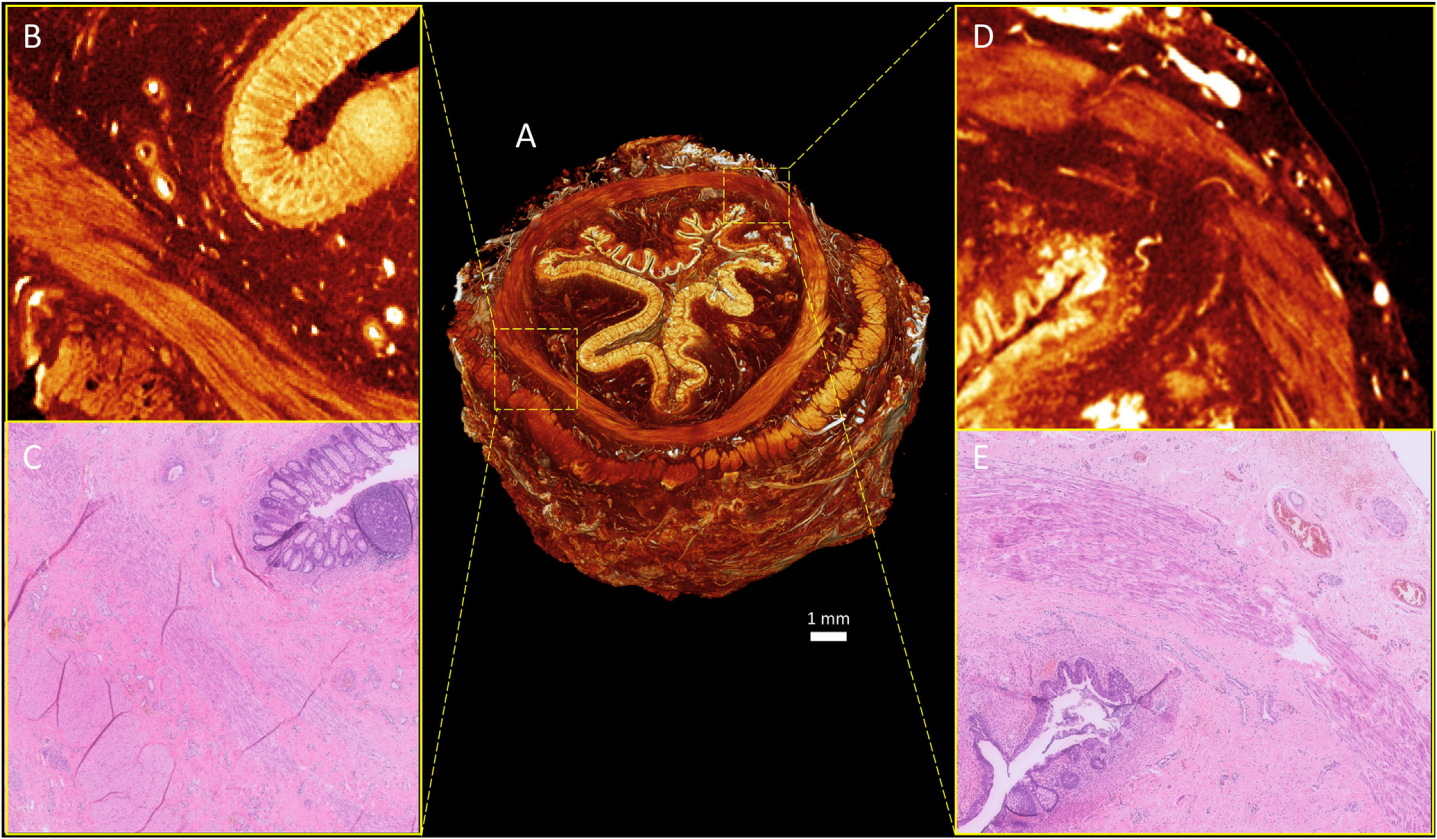
Comparison of areas within one sample. (A) Micro-CT Rendering of ARM 6 with axial slice, revealing the muscle layers and lumen. Areas of interest have been highlighted that correspond with panel B and C or D and E respectively. (B) Zoomed in section in micro-CT scan revealing healthy looking muscle fibers in an undamaged part of the sample of both the longitudinal and circular muscle layer. (C) Histological slide of same area as B. (D) Zoomed in section in micro-CT scan of area close to resection margin that appears to be damaged as indicated by hematomas and disarrangement of muscle fibers. (E) Histological section corresponding to D. Surgical margin is closer to the circular layer in this part of the sample and no longitudinal fibers can be seen. Micro-CT offers imagery of the full sample, allowing for area selection to do correlative imaging to get a representable sample.

Histological examination was previously the method of choice to chart the fistula in ARM. Although histology is able to investigate structures in more detail than micro-CT, and there is vast experience using this technique, its limitation is that it investigates a fragment of the entire specimen in a 2-dimensional plane. Findings that could aid in our understanding of a disease could be hiding a little more proximal, caudal, left or right from where the histological sample is taken (Figure 5). Using micro-CT imaging complementary to histology, for the first time we were able to image and analyze full samples in 3-dimensional at microscale resolution to provide new insights of the ARM fistula.

The 3-dimensional nature of micro-CT imaging allows for new ways of interpreting the microscale structures in tissue samples. Therefore we were able to distinguish the IAS in these fistulas which was previously not possible. This insight provides new arguments in the discussion on whether or not the fistula should be preserved during surgery. The protocol used here is adaptable to other kinds of tissue and research questions that could benefit from analyzing microscale structures in 3-dimensional in a relatively quick and nondestructive fashion.

### Strengths and Implications

Micro-CT imaging can visualize the tissue composition within a full ex-vivo sample of multiple centimeters and provides an image that is comparable to histology. Micro-CT imaging could be used in the future to better study dissection planes in different surgical specialties or even to guide pathological assessment in a clinical setting.

This research indicates that the fistula is in fact an ectopic anus with all elements present. Although we cannot conclude on its functionality in this research, it indeed questions why we resect the fistula during the ASARP or PSARP. By relocating the fistula toward the external sphincter, it becomes too long for anoplasty and could lead to anorectal prolapse.^24^ Simply leaving the fistula intact and relocating it could increase the incidence of this complication and might call for alternative surgical approaches, such as securing the rectum to the presacral fascia. If one would preserve the fistula, the current margins taken are too slim to encompass the whole IAS for relocating. In all our samples, damage occurred to the structures such as the muscle layers due to margins being close to them. Proximally, typically the margins moved more laterally as the dissection plane diverted from the adhering structure and more of the fistula could be resected. If one wishes to relocate the entire fistula without resecting its distal part, ideally wider margins would be employed to spare the IAS. This is a challenge in itself, as adjacent structures need to be spared that are in close proximity in for example rectovestibular cases, where the anterior margin consist of mucosa, or even more difficult in rectourethral fistulas. The recently developed Posterial Rectal Advancement Anoplasty for rectoperineal fistulas that partly reside within the external sphincter does not involve any resection of the distal end.^25^^,^^26^ The data we provide here supports fistula sparring techniques like the Posterial Rectal Advancement Anoplasty as it demonstrates normal morphology. Future studies will need to expand our knowledge on the functionality of the fistula and the practical aspects of fistula sparring procedures in other forms of ARM.

### Limitations

The low number of samples was a limiting factor for this research. Moreover, all but one sample were rectovestibular or rectoperineal fistulas. Thus, this research cannot yet be extrapolated to high ARMs such as rectovesical, prostatic, or vaginal fistulas and pouch colons or cloacas. The control sample used in this study was a fetal sample and therefore not fully developed. Although this sample was picked to be least likely to have an abnormal anal canal, it still had a genetic abnormality (22q11 deletion) as the reason for the termination of pregnancy.

Micro-CT is not validated for analyzing clinical samples. The analyses made here are limited to the assessment of our expert panel. In this study we attempted to overcome this limitation by histology as verification method, with similar results, indicating that micro-CT could be suitable for analyzing samples of this kind and with more research and experience promises to be a valuable tool for clinical research.

## Conclusion

Using micro-CT imaging, we have shed new light on fundamental ARM morphology and highlight this innovative technique for biomedical and surgical research. Through this technique, we found indications that the fistula in ARM patients contains the elements of an anal canal and might be considered an ectopic anus. By removing the fistula during surgery, the IAS is removed and thus an important element contributing to fecal continence disappears. Saving and replacing the fistula during ARM surgery could save the IAS and might improve continence in ARM patients. Future studies should focus on determining if this ectopic anal canal is functional, possibilities of preserving surgical techniques and the impact thereof on patient reported outcomes.
